# Survival analysis of patients with tuberculosis and risk factors for multidrug-resistant tuberculosis in Monrovia, Liberia

**DOI:** 10.1371/journal.pone.0249474

**Published:** 2021-04-23

**Authors:** Boye Bobby Carter, Yang Zhang, Hangjin Zou, Chuhan Zhang, Xinming Zhang, Rongtian Sheng, Yanfei Qi, Changgui Kou, Yin Li

**Affiliations:** School of Public Health, Jilin University, Changchun, Jilin, China; Jamia Hamdard, INDIA

## Abstract

We reviewed the records of 337 confirmed cases of tuberculosis patients in Monrovia, the capital of Liberia, 2015. The risk factors affecting the survival and multidrug-resistance of tuberculosis patients were examined. Kaplan-Meier analysis and the log-rank test were used to assess the differences in survival among the patients, while Cox regression model was used for multivariate analysis. The qualitative data was tested with chi-square test in the single factor analysis of multidrug-resistant TB. Multivariate analysis was performed using binary logistic regression analysis. The significance level for all the tests were set at 0.05. The mean period of the follow-up of patients was 10 months. In the 337 patients, 33 (9.8%) died, the 21-month survival rate was 90.2%. The results of multivariate Cox regression analysis show that overcrowding (HR = 7.942, 95% CI 3.258–19.356), former smoking (HR = 3.773, 95% CI 1.601–8.889), current smoking (HR = 3.546, 95% CI 1.195–10.521), multidrug-resistance tuberculosis (HR = 4.632, 95% CI 1.913–11.217) were risk factors for death during anti-tuberculosis treatment in TB patients in Liberia. The results of binary logistic regression analysis show that extra-pulmonary (OR = 2.032, 95% CI 1.133–3.644), family history of TB (OR = 2.387, 95% CI 1.186–4.807) and current smoking (OR = 3.436, 95% CI 1.681–7.027) were risk factors for multidrug-resistant tuberculosis. These results can provide insights on local tuberculosis early intervention, increase public health awareness, and strengthen the control of factors that may affect the survival and multidrug-resistance of tuberculosis patients.

## Introduction

Tuberculosis (TB) is one of the top 10 causes of death worldwide. In 2018, 10 million people fell ill with TB worldwide and 1.5 million people lost their lives to the disease [1, 2]. A quarter of the world’s population is estimated to be infected with TB bacteria and is thus at risk of developing the disease [1, 2]. According to WHO estimates in 2018, the number of new tuberculosis cases in Africa accounted for 24% of the world’s total death, the second largest to Southeast Asia (44%) [1].

In 2018, 87% of new TB cases occurred in the 30 high TB burdened countries [3]. Liberia-Africa’s oldest country, is among the thirty high TB burdened countries in the world [1]. WHO estimated the incidence rate for all forms of tuberculosis in Liberia at 308 per 100,000 population, more than twice the global average of 133 per 100,000 population [1]. The trend of the country’s TB prevalence has been on the increase since 1990, although prevalence began leveling off since 2010, it has nonetheless maintained an upward trajectory [4].

Multidrug-resistant tuberculosis (MDR-TB) is a form of TB caused by mycobacterium tuberculosis (MTB) that do not respond to isoniazid and rifampicin, the two most powerful first-line anti-TB drugs [1]. In the process of TB control worldwide, MDR-TB continues to be an increasingly important issue. MDR-TB may be due to primary infection acquired or developed in the course of treatment [5]. Compared with drug-susceptible TB, multi-resistant TB usually requires a longer treatment cycle with poorer treatment results and a higher risk of death [6]. There may also be more severe drug resistance during the treatment of TB. According to data reported by WHO in 2018, the success rate of treatment for MDR-TB is 56% globally [1]. The treatment of MDR-TB is extremely expensive and consumes a large proportion of medical resources [7].

In this work, we investigated the possible risk factors affecting the survival of 337 TB patients confirmed cases of tuberculosis patients in Monrovia 2015. We also estimates of the risk factors of MDR-TB patients.

## Materials and methods

### Collection of data

Using retrospective research methods, we reviewed the records of 337 patients diagnosed and treated for TB at National Leprosy and Tuberculosis Control Program in Monrovia, Liberia in 2015 [8]. Chest X-ray, GeneXpert MTB/RIF and smear microscopy are used to diagnose tuberculosis patients. GeneXpert MTB/RIF and Mycobacterium tuberculosis drug sensitivity test were used in present study to find out MDR-TB patients. The death of tuberculosis patients was defined as the event result of this study. Patients with non-event results were defined as censored data, including patients who were cured, failed treatment, and lost to follow-up. The patient’s survival time was calculated from the date of diagnosis of tuberculosis to the date of cure, last follow-up or death. Use door-to-door surveys, letters or home phone follow-ups to obtain information about the patient’s survival status. All patients were followed up until June 2017. The study included only patients that were 14 years old and above at the time of diagnosis and treatment in 2015. Data were collected through the National Tuberculosis Notification form developed by Ministry of Health and Social Welfare in Liberia. The form which is a 25-items questionnaire was divided into 6-sections, namely Demographic information, Clinical characteristics, Radiological details, Mycobacteriology characteristics, risk factors, and treatment outcome.

### Definition of variables

Overcrowding was defined as two or more adult of the same gender sharing the same room [9]. Tobacco smoking was defined as current or former use of at least ten cigarettes in a month. We defined history of TB as either mother or father having or had tuberculosis. Multidrug- resistant TB was defined as resistance to at least isoniazid and rifampicin, requiring the use of second-line drugs [10]. Non-MDR-TB included drug-susceptible TB, mono resistance TB and poly drug resistant TB. Depending on whether rifampin-resistant or sensitive, the treatment was started.

### Data analysis

Data were entered into an access database using Epidata software (Version 3.1, Odense, Denmark), cleaned and analyzed using the statistical package for the social sciences (SPSS, version 24.0, IBM SPSS, IBM Corp, Armonk, NY, USA). Kaplan-Meier analysis and the log-rank test were used to assess the differences in survival among patients. Cox regression model was used for multivariate analysis. In the single factor analysis of MDR-TB, the qualitative data was tested with chi-square test, multivariate analysis was performed using binary logistic regression analysis, and the significant level for all the tests was set at 0.05.

### Ethics consideration

Patients in this study were fully anonymized before accessing their files. A letter was written by the Ministry of Health and Social Welfare, Liberia to the National Leprosy and Tuberculosis Control Program (NLTCP) administrator for permission to access the raw data. Before the data collection, written consent was obtained from the heads of each department of the hospital and confidentiality was maintained by coding from data collection to analysis. Each patient was assigned a unique study number for identification. Patients’ names were not used on any study related documents.

## Results

### Description of research subject

**Fig 1:**
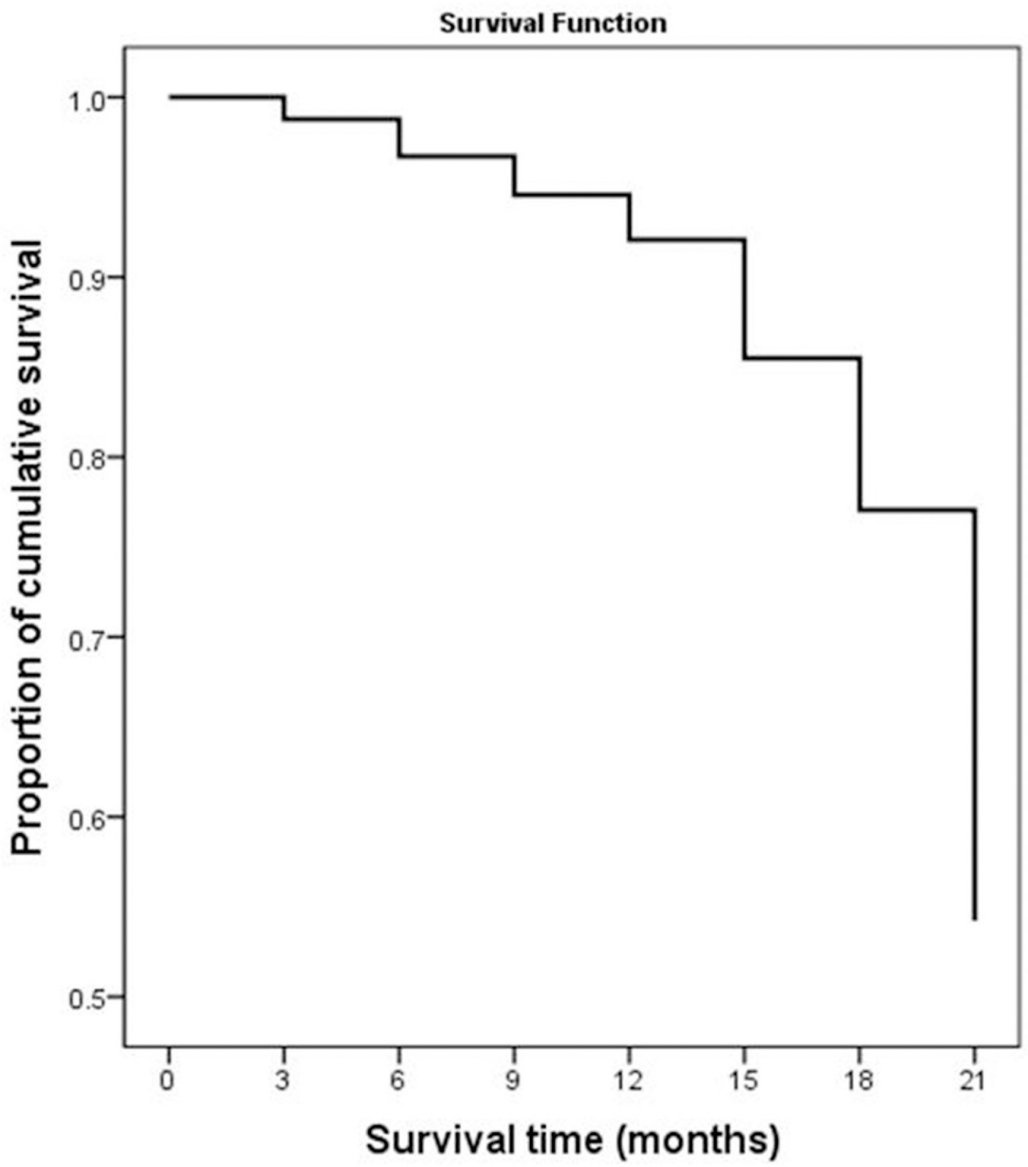
21-month cumulative survival rate of tuberculosis patients in Liberia.

Basic demographics and clinical characteristics of patients with TB are presented in Table 1. Male and female patients were 257 (76.3%) and 23.7% (80/337), respectively. The ages of patients ranged from 14 to 67 years old. 98.5% (332/337) of TB patients were 18 years or older. 74.8% (252/337) patients were engaged in heavy physical labor (carpenters, farmers, fishermen, plumbers or marketers). While, 25.2% (85/337) patients were accountants, administrators, pastors, teachers or students. More than half of TB patients, 52.2% (176/337) suffered from HIV-Aids. Nearly three quarters of TB patients 250 (74.2%) had constitutional TB symptoms. During the follow-up period, 33 out of 337 cases, 9.8%, died. Among the 337 patients, 129 (38.3%) cases were MDR-TB patients. Table 2 shows the survival data for all patients with TB. The median survival time and cumulative probability of survival were 18 and 54%, respectively, as shown in Fig 1 and Table 2.

**Table 1:** Demographic and clinic characteristics of TB patients in Liberian (n = 337).

Variables	Groups	Number (n)	Constituent Ratio (%)
Gender	Males	257	76.3
	Females	80	23.7
Age	14~	5	1.5
	18~	151	44.8
	36~	133	39.5
	54~67	48	14.2
Nationality	Liberian	324	96.1
	The others	13	3.9
Occupation	Heavy physical labor	252	74.8
	The others	85	25.2
Education level	Not educated	45	13.4
	Junior middle school	165	49.0
	Senior high school	99	29.4
	College or higher	28	8.3
Marital status	Single/Divorce/Widowhood	279	82.8
	Married	58	17.2
Overcrowding	No	310	92.0
	Yes	27	8.0
Poverty-stricken	No	55	16.3
	Yes	282	83.7
Smoking	Never	168	49.9
	Former	121	35.9
	Current	48	14.2
Drinking	No	48	14.2
	Yes	289	85.8
Family History	No	290	86.1
	Yes	47	13.9
BMI	Normal (18.5–24)	114	33.8
	Below normal (<18.5)	109	32.3
	Above normal (≥24)	114	33.8
Comorbidity	None	38	11.3
	HIV-Aids	176	52.2
	Lung Cancer	90	26.7
	The others	33	9.8
Drug sensitivity	Any drug resistance	208	61.7
	Multi-drug resistance	129	38.3
Chest X-ray Findings	Cavities	57	16.9
	Parenchymal	239	70.9
	Pleural	41	12.2
Constitutional symptoms	No	87	25.8
	Yes	250	74.2
Site of TB	Pulmonary	271	80.4
	Extra Pulmonary	66	19.6
Treatment of TB	Initial treatment	273	81.0
	retreatment	64	19.0
Death	No	304	90.2
	Yes	33	9.8

**Table 2:** Life table for the TB patients in Liberian (n = 337).

Time (months)	Number entering interval	Number exposed to risk	Cumulative proportion surviving	Survival standard error
0	337	327	99%	0.01
3	312	287	97%	0.01
6	255	226	95%	0.01
9	192	152	92%	0.02
12	108	84	86%	0.03
15	54	41	77%	0.05
18	23	14	54%	0.10

### Survival analysis of TB patients

**Fig 2:**
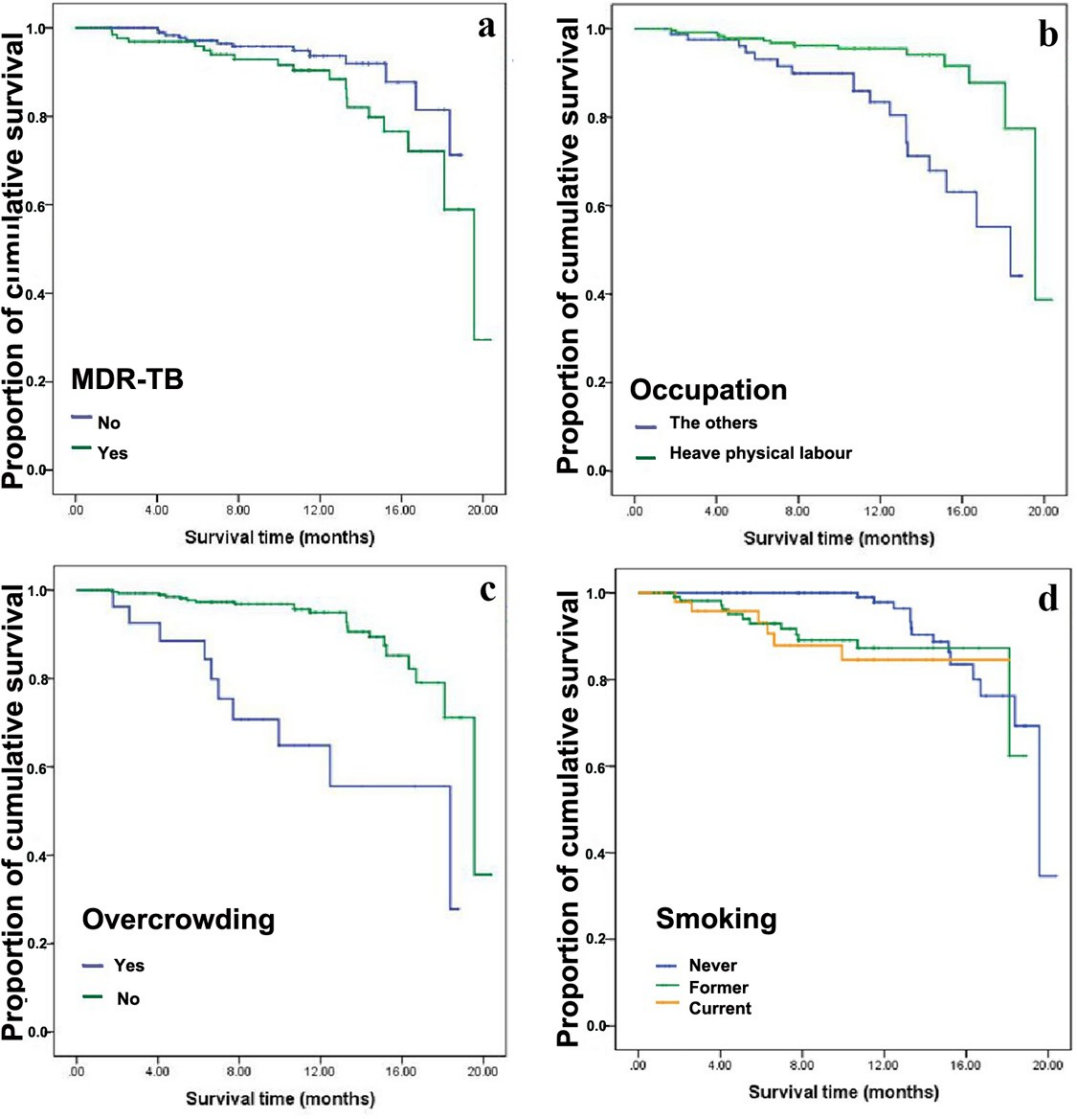
21-months cumulative probability of survival of patients with TB according to whether overcrowding, whether MDR-TB, occupation and whether smoking in Monrovia, Liberia (2015). a. cumulative probability of survival according to whether MDR-TB, b. cumulative probability of survival according to occupation, c. cumulative probability of survival according to whether overcrowding, d. cumulative probability of survival according to whether smoking.

Kaplan-Meier survival function test was used to estimate the cumulative probability of survival of different gender, marital status, religion, overcrowding, MDR-TB, occupation, CXR and smoking. The results of log-rank test show that the differences of cumulative probability of survival of various factors such as gender, marital status, overcrowding, MDR-TB, occupation, CXR and smoking are statistically significant (*p* < 0.05), shown in Fig 2 and Table 3.

**Table 3:** The Log-rank test of factors influencing survival of TB patients in Liberian. (n = 337).

Variables	Categories	Total	Died (%)	Chi-square	*P*-value
Gender	Males	257	20 (7.8)	6.593	0.010
	Females	80	13 (16.2)		
Marital status	Married	58	0	5.282	0.022
	Single	279	33 (11.8)		
Overcrowding	Yes	27	10 (37.0)	22.220	<0.001
	No	310	23 (7.4)		
Occupation	Heavy physical labor	252	15 (6.0)	14.990	<0.001
	The others	85	18 (21.2)		
MDR-TB	Yes	129	20 (15.5)	4.014	0.045
	No	208	13 (6.2)		
Smoking	Never	168	14 (8.3)	6.147	0.046
	Former	121	13 (10.7)		
	Current	48	6 (12.5)		
Chest X-ray	Cavities	57	11 (19.3)	10.640	0.005
	Parenchymal	239	20 (8.4)		
	Pleural	41	2 (4.9)		

**Fig 3:**
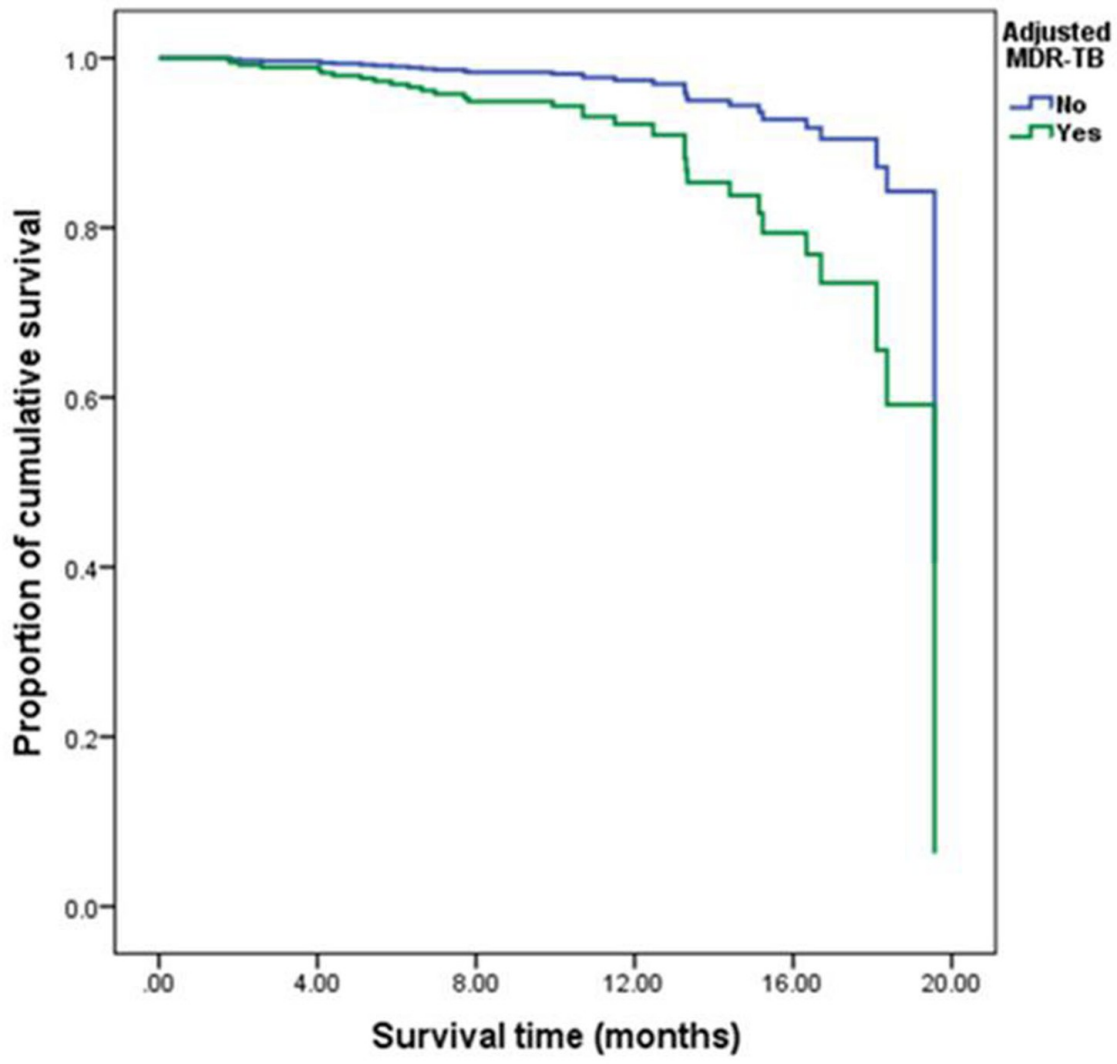
Comparison of cumulative survival function of patients with MDR-TB and patients with non-drug-resistant tuberculosis after controlling other covariables including overcrowding, occupation and smoking.

After controlling of age factor, several statistically significant factors (*p* < 0.05) of Kaplan-Meier univariate analyses including gender, marital status, overcrowding, MDR-TB, occupation, CXR and smoking were included in the Cox regression model for multivariate analysis. According to the multivariate analysis, former smoking (HR 3.773, 95% CI 1.601–8.889, *p* = 0.002), current smoking (HR 3.546, 95% CI 1.195–10.521, *p* = 0.023), overcrowding (HR 7.942, 95% CI 3.258–19.356, *p <*0.001) and MDR-TB (HR 4.632, 95%CI 1.913–11.217, *p* = 0.001) were all associated with increased risk of death during TB treatment in the 337 patients (Table 4). The survival status of Non-MDR-patients were better than that of MDR-TB patients (Fig 3).

**Table 4:** Multivariable Cox regression of factors influencing survival of TB patients in Liberia (n = 337).

Variables	*β*	*S*.*E*.	*Wald χ*^2^	*P-value*	HR (95%CI for HR)
Occupation					
The others					1.000
Heavy physical	-1.060	0.400	7.030	0.008	0.347 (0.158–0.759)
Overcrowding					
No					1.000
Yes	2.072	0.455	20.782	<0.001	7.942 (3.258–19.356)
Smoking					
Never					1.000
Former	1.328	0.437	9.219	0.002	3.773 (1.601–8.889)
Current	1.266	0.555	5.202	0.023	3.546 (1.195–10.521)
MDR-TB					
No					1.000
Yes	1.533	0.451	11.541	0.001	4.632 (1.913–11.217)

### Analysis of the risk factors of MDR-TB

To investigate the risk factors affecting the occurrence of MDR-TB patients in Liberia, a univariate analysis was performed on TB patients admitted in 2015. As shown in Table 5, the differences in the incidences of MDR-TB patients among different categories were statistically significant in the marital status, smoking, site of TB, family history and constitutional symptom factors (*p* <0.05).

**Table 5:** Comparison of MDR-TB and Non-MDR-TB patients in Liberia (n = 337).

Variables	Categories	Total	MDR-TB (%)	Non-MDR-TB	Chi-square	*P*-value
Marital status	Single	279	114 (40.9)	168	4.572	0.038
	Married	58	15 (25.9)	43		
Smoking	Never	168	63 (37.5)	105	15.686	<0.001
	Former	121	36 (29.8)	85		
	Current	48	30 (62.5)	18		
Site of TB	Pulmonary	271	97 (35.8)	174	3.618	0.067
	Extrapulmonary	66	32 (48.5)	34		
Family History	No	290	102 (35.2)	188	8.493	0.006
	Yes	47	27 (57.4)	20		
Constitutional symptom	No	87	50 (57.5)	37	18.284	<0.001
	Yes	250	79 (31.6)	171		

After controlling of gender and age factors, five statistically significant factors including marital status, smoking, site of TB, family history and constitutional symptom (*p<*0.05) were calculated in the binary multivariable logistic regression analysis. As shown in Table 6, extra-pulmonary (OR 2.032, 95% CI 1.133–3.644, *p* = 0.017), family history of TB (OR 2.387, 95%CI 1.186–4.807, *p* = 0.015) and current smoking (OR 3.436, 95% CI 1.681–7.027, *p* = 0.001) were risk factors for MDR-TB patients.

**Table 6:** Predictors of MDR-TB patients registered for treatment in Liberia in 2015 (n = 337).

Covariables	*β*	*S*.*E*.	*Wald χ*^2^	*P-value*	OR (95%CI for OR)
Constant	-0.133	0.306	0.188	0.665	0.876
Site of TB					
Pulmonary					1.000
Extrapulmonary	0.709	0.298	5.656	0.017	2.032 (1.133–3.644)
Family history					
No					1.000
Yes	0.870	0.357	5.938	0.015	2.387 (1.186–4.807)
Smoking					
Never					1.000
Former	-0.260	0.283	0.839	0.360	0.771 (0.443–1.344)
Current	1.234	0.365	11.440	0.001	3.436 (1.681–7.027)
Constitutional symptom					
No					1.000
Yes	-0.987	0.272	13.178	<0.001	0.373 (0.219–0.635)

## Discussion

The multivariate analysis showed that former smoking (HR 3.773, 95% CI 1.601–8.889, *p* = 0.002) and current smoking (HR 3.546, 95% CI 1.195–10.521, *p* = 0.023) were all associated with increased risk of death in TB patients. The strong relation between smoking and TB were also reported in the literature [11, 12]. In 2018, 0.86 million new TB cases worldwide were attributable to smoking, tobacco smoking increased the risk of TB disease by a factor of 1.6 [1]. Our finding is similar to a recent study result which revealed that compared with nonsmokers, those who smoke tobacco have twice the risk of TB disease [13, 14]. Cigarette smoking independently increases the risk of death for TB patients [15], and patients with TB who smoke have nearly twice the risk of death during TB treatment [16]. TB patients who were non-smokers were discovered to have higher cure rates when compared with TB patients who were smokers [12]. An estimated 15.2% (95% CI 1.8–31.9) of TB mortality was attributable to smoking [3]. This could be due to fact that current and former smokers might develop a respiratory symptom that may lead to lung cancer, coronary artery disease or chronic bronchitis [17]. Currently, there are more than 1 billion tobacco users, nearly 80% of whom live in low- and middle-income countries (LMIC), where there are limited available health resources to manage costly diseases including TB [3, 18]. However, an improved understanding of the contribution of smoking to TB disease and TB mortality is needed to better characterize the joint impact of smoking and TB on health burdens in LMIC [3].

TB patients living in overcrowded areas were 7.942 times more likely to die than patients living in non-overcrowded areas (HR 7.942, 95% CI 3.258–19.356; *p* <0.001). There remains limited research on the association between TB mortality rate and population residence. However, poverty, unemployment and illiteracy can affect access and the utilization of health care services and subsequently affect the outcome of TB [5]. A study published in 2011 from South Africa reported that poor housing quality and overcrowding were significantly associated with increased prevalence of TB [19]. In addition, drug resistant strains of M. tuberculosis may be transmitted in the crowded community.

According to the univariate analysis, our result found that females had higher risk of death from TB (16.2%) than males (7.8%). This finding correlates with similar studies among hospitalized pulmonary TB patients in Bolivia and Erbil which reported that after controlling for other variables, including coexisting pathology and extent of lung disease, females were found to be more likely to die from TB than males [20], On the contrary, a study conducted by Martinez Rodríguez et al. showed that survival was significantly lower among men compared to women [21]. Studies in Iraqi Kurdistan showed that no significant difference between male and female patients’ in terms of mortality. Multivariate analysis showed that gender factors in our study did not affect the survival of TB patients [8, 22]. The difference in these findings requires further studies with regards to factors responsible for the difference in survival between males and females.

MDR-TB (HR 4.632, 95% CI 1.913–11.217, *p =* 0.001) was identified as a risk factor for death during anti-tuberculosis treatment in TB patients. This result correlates with previous findings in Peru [6]. In 2015, 30% of the 3.4 million new bacteriologically confirmed and previously treated TB cases notified globally were reported to have had MDR-TB [18]. Of the subjects in this study, 129 (38.3%) were MDR-TB patients, which was higher than that of the global average for that year. It indicates that in Liberia there may be high rates of acquired resistance to anti-TB medications. Schematically, MDR-TB could be from inappropriate prescription of anti-TB treatment regimens, inadequate drug supply, poor quality of drugs, high default and treatment failure rates [23, 24]. Furthermore, public health systems in low-and middle-income countries often have weak infrastructure and insufficient resources that may increase the risk of death from drug-resistant tuberculosis [25, 26]. The TB burden in Liberia has been driven by disruption of TB control services during the civil war. Stock outs have been experienced due to poor supply chain management. During the post war period efforts have been made to control TB but accessibility and patient awareness are still inadequate.

Since MDR-TB had an important impact on patient survival compared to non-MDR-TB, we analyzed the risk of developing MDR-TB. Studies from India have reported that the prevalence of MDR-TB is higher in EPTB cases, although it has not been compared with pulmonary tuberculosis [27, 28]. According to the multivariate analysis, extra-pulmonary tuberculosis could increase risk of acquiring MDR-TB (OR 2.032, 95% CI 1.133–3.644, *p* = 0.017). We also found that patients with a family history of TB were 2.387 times more likely to develop multidrug resistance (OR 2.387, 95% CI 1.186–4.807, *p =* 0.015). A study in Mali has confirmed that a history of contact with TB patients could significantly increase the risk of being diagnosed with MDR-TB disease [29]. Patients who maintained smoking habits were 3.436 times more likely to develop MDR-TB than those never smoking (OR 3.436, 95% CI 1.681–7.027, *p* = 0.001). Cigarette smoking adversely affects culture conversion during anti-tuberculosis treatment, which extends treatment cycles [30]. Prolonged infection may lead to additional transmission of MDR-TB [31]. Resistance could also be due to continual and inappropriate administration of medication that made the bacteria mutate and develop resistance against the drugs. We could not analyze these factors due to the lack of relevant data. Detailed studies are needed to address this question. Additionally, effective implementation of the directly observed treatment strategy and increasing number of institutions equipped with drug resistance tests for early detection of primary resistance are mandatory.

## Conclusions

In conclusion, overcrowding, smoking, MDR-TB were important risk factors and negatively affected the survival rates of TB patients in Liberia. At the same time, extra-pulmonary, family history of TB and current smoking were significant risk factors and negatively affected the survival rates of MDR-TB patients in Liberia. The results suggested that the mycobacterium tuberculosis drug sensitivity test should be strengthened. In addition, the genotype DST method should also be considered to determine the prevalence of drug-resistant tuberculosis, so as to detect patients with MDR-TB more sensitively and early.

## Supporting information

S1 Dataset (XLS)

S1 File The survey questions and minimal data are in the supporting information part. (DOCX)
