# Supplementary material for: Survival analysis of patients with tuberculosis and risk factors for multidrug-resistant tuberculosis in Monrovia, Liberia
Source: PLoS One. 2021 Apr 23;16(4):e0249474. doi: 10.1371/journal.pone.0249474 (PMC8064579; doi:10.1371/journal.pone.0249474)
Supplement: S1 File — (DOCX) [file pone.0249474.s002.docx]

**S1 File. Survival analysis of patients with tuberculosis and risk factors for multidrug-resistant tuberculosis in Monrovia, Liberia**

Boye Bobby Carter^¶^, Yang Zhang^¶^, Hangjin Zou^&^, Chuhan Zhang^&^, Xinming Zhang^&^, Rongtian Sheng^&^, Yanfei Qi^*^, Changgui Kou^*^, Yin Li^*^

School of Public Health, Jilin University, Changchun, Jilin, China

**QUESTIONNAIRES**

1. **Demographic information**

**Age:**

**Gender:** Male ( ) Female ( )

**Nationality:** Guinean ( ) Liberian ( ) Ghanaian ( ) Sierra Leonean ( )

Nigerian ( )

**Religion:** Christian ( ) Muslim ( )

**Occupation:** please specify

**Education:** Not educated ( ) Primary level ( ) Secondary education ( ) University degree ( )

**Marital Status**: Married ( ) Single ( )

**Present address:**

**Phone:**

1. **Risk factors**

**Smoking History (current or former use of at least ten cigarettes in a month):** Never Smoke ( ) Former Smoke ( ) Current Smoker ( )

**Alcohol:** Yes ( ) No ( )

**Family history of TB (mother or father having or had tuberculosis):**

Yes ( ) No ( )

**Overcrowding (two or more adult of the same gender sharing the same room):** Yes ( ) No ( )

**Below Poverty Line:** Yes ( ) No ( )

1. **Clinical characteristics**

**Weight:** **Height**:

**Treatment Category**: Initial treatment ( ) Retreatment ( )

**Drug Therapy**:

**TB symptom**:

**Comorbidity:** HIV ( ) None ( ) Lung Cancer ( ) any other specify **4. Radiological details**

**Site of tuberculosis**: Pulmonary ( ) Extra Pulmonary ( )

**Chest X-ray Findings:** Parenchymal ( ) Cavities ( ) Pleural ( )

1. **Micro bacteriology characteristics**

**Smear:** Negative ( ) Positive ( )

**Culture and drug sensitivity**: Any drug resistance ( ) Multi drug resistance ( )

1. **treatment outcome**

Complete cured ( ) Cured ( ) On treatment ( ) Died ( )

Treatment defaulted ( )
